# Pure discrete spectrum and regular model sets in *d*-dimensional unimodular substitution tilings

**DOI:** 10.1107/S2053273320009717

**Published:** 2020-08-21

**Authors:** Dong-il Lee, Shigeki Akiyama, Jeong-Yup Lee

**Affiliations:** a Department of Mathematics, Seoul Women’s University, Seoul 01797, Republic of Korea; b Institute of Mathematics, University of Tsukuba, 1-1-1 Tennodai, Tsukuba, Ibaraki 305-8571, Japan; c Department of Mathematics Education, Catholic Kwandong University, Gangneung 25601, Republic of Korea; d KIAS, 85 Hoegiro, Dongdaemun-gu, Seoul, 02455, Republic of Korea

**Keywords:** Pisot family substitution tilings, pure discrete spectrum, regular model sets, Meyer sets, rigidity

## Abstract

Primitive substitution tilings on  whose expansion maps are unimodular are considered. It is assumed that all the eigenvalues of the expansion maps are algebraic conjugates with the same multiplicity. In this case, a cut-and-project scheme can be constructed with a Euclidean internal space. Under some additional condition, it is shown that if the substitution tiling has pure discrete spectrum, then the corresponding representative point sets are regular model sets in that cut-and-project scheme.

## Abstract 2

The equivalence between pure discrete spectrum and regular model sets in *d*-dimensional unimodular substitution tilings is discussed.

## 1. Introduction   

In the study of aperiodic tilings, it has been an interesting problem to characterize pure discrete spectrum of tiling dynamical systems (Baake & Moody, 2004 ▸). This property is related to understanding the structure of mathematical quasicrystals. For this direction of study, substitution tilings have been good models, since they have highly symmetrical structures. A lot of research has been done in this direction (see Akiyama *et al.*, 2015 ▸; Baake & Grimm, 2013 ▸ and references therein). Given a substitution tiling with pure discrete spectrum, it is known that this can be described via a cut-and-project scheme (CPS) (Lee, 2007 ▸). However, in the work of Lee (2007 ▸), the construction of the CPS is with an abstract internal space built from the pure discrete spectral property. Since the internal space is an abstract space, it is neither easy to understand the tiling structure, nor clear if the model sets are regular or not. In the case of one-dimensional substitution tilings with pure discrete spectrum, it is shown that a CPS with a Euclidean internal space can be built and the corresponding representative point sets are regular model sets (Barge & Kwapisz, 2006 ▸). In this paper, we consider substitution tilings on 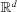 with pure discrete spectrum whose expansion maps are unimodular. We show that it is possible to construct a CPS with a Euclidean internal space and that the corresponding representative point sets are regular model sets in that CPS.

The outline of the paper is as follows. First, we consider a repetitive primitive substitution tiling on 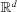 whose expansion map is unimodular. Then we build a CPS with a Euclidean internal space in Section 3. In Section 4, we discuss some known results around pure discrete spectrum, Meyer set and Pisot family. In Section 5, under the assumption of pure discrete spectrum, we show that each representative point set of the tiling is actually a regular model set in the CPS with a Euclidean internal space.

## 2. Preliminaries   

### 2.1. Tilings   

We begin with a set of types (or colours) 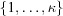, which we fix once and for all. A *tile* in 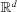 is defined as a pair 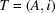 where 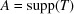 (the support of *T*) is a compact set in 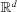, which is the closure of its interior, and 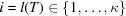 is the type of *T*.

We let 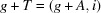 for 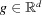. We say that a set *P* of tiles is a *patch* if the number of tiles in *P* is finite and the tiles of *P* have mutually disjoint interiors. The *support of a patch* is the union of the supports of the tiles that are in it. The *translate of a patch P* by 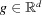 is 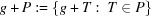. We say that two patches 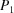 and 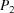 are *translationally equivalent* if 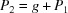 for some 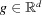. A *tiling* of 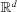 is a set 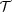 of tiles such that 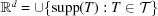 and distinct tiles have disjoint interiors. We always assume that any two 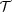-tiles with the same colour are translationally equivalent (hence there are finitely many 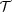-tiles up to translations). Given a tiling 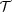, a finite set of tiles of 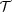 is called a 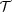-*patch*. Recall that a tiling 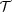 is said to be *repetitive* if every 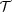-patch occurs relatively densely in space, up to translation. We say that a tiling 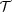 has *finite local complexity* (FLC) if, for every *R* > 0, there are finitely many equivalence classes of 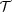-patches of diameter less than *R*.

### 2.2. Delone κ-sets   

A κ-*set* in 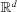 is a subset 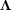 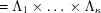 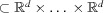 (κ copies) where 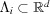 and κ is the number of colours. We also write 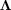 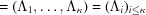. Recall that a Delone set is a relatively dense and uniformly discrete subset of 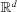. We say that 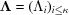 is a *Delone* κ-*set* in 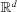 if each 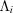 is Delone and 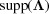 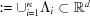 is Delone.

The types (or colours) of points for Delone κ-sets have a meaning analogous to the colours of tiles for tilings. We define repetitivity and FLC for a Delone κ-set in the same way as for tilings. A Delone set Λ is called a *Meyer set* in 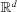 if 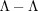 is uniformly discrete, which is equivalent to saying that 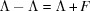 for some finite set *F* (see Moody, 1997 ▸). If 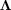 is a Delone κ-set and 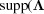) is a Meyer set, we say that 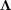 is a Meyer set.

### 2.3. Substitutions   

We say that a linear map 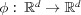 is *expansive* if there is a constant *c* > 1 with 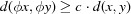
for all 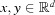 under some metric *d* on 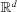 compatible with the standard topology.

**Definition 2.1**

Let 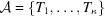 be a finite set of tiles on 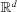 such that 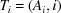; we will call them *prototiles*. Denote by 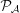 the set of patches made of tiles each of which is a translate of one of 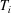
’s. We say that 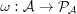 is a *tile-substitution* (or simply *substitution*) with an expansive map ϕ if there exist finite sets 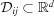 for 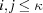, such that 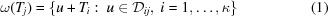
with 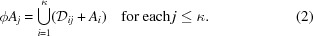
Here all sets in the right-hand side must have disjoint interiors; it is possible for some of the 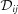 to be empty. We call the finite set 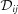 a *digit set* (Lagarias & Wang, 1996 ▸). The *substitution* 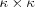 *matrix* 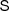 of the tile-substitution is defined by 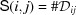. We say that ϕ is *unimodular* if the minimal polynomial of ϕ over 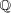 has constant term 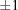 (*i.e.* 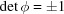); that is to say, the product of all roots of the minimal polynomial of ϕ is 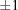.

Note that for 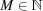


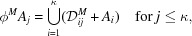
where 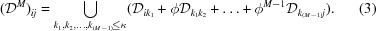
The tile-substitution is extended to translated prototiles by 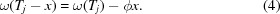
The equations (2) allow us to extend ω to patches in 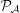 defined by 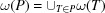. It is similarly extended to tilings all of whose tiles are translates of the prototiles from 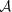. A tiling 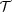 satisfying 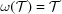 is called a *fixed point of the tile-substitution*, or a *substitution tiling with expansion map* ϕ. It is known that one can always find a periodic point for ω in the tiling dynamical hull, *i.e.* 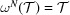 for some 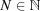. In this case we use 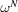 in the place of ω to obtain a fixed-point tiling. We say that the substitution tiling 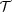 is *primitive*, if there is an 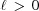 for which 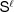 has no zero entries, where 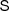 is the substitution matrix.

**Definition 2.2**

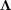
 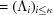 is called a *substitution Delone* κ-*set* if 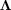 is a Delone κ-set and there exist an expansive map 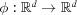 and finite sets 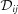 for 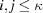 such that 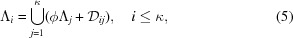
where the unions on the right-hand side are disjoint.

There is a standard way to choose distinguished points in the tiles of a primitive substitution tiling so that they form a ϕ-invariant Delone κ-set. They are called *control points* (Thurston, 1989 ▸; Praggastis, 1999 ▸) which are defined below.

**Definition 2.3**

Let 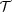 be a fixed point of a primitive substitution with an expansion map ϕ. For every 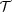-tile *T*, we choose a tile 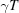 on the patch 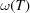; for all tiles of the same type, we choose 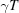 with the same relative position. This defines a map 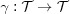 called the *tile map*. Then we define the *control point* for a tile 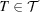 by 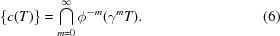

The control points satisfy the following:

(*a*) 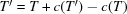, for any tiles 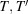 of the same type;

(*b*) 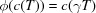, for 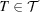.

For tiles of any tiling 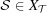, the control points have the same relative position as in 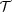-tiles. The choice of control points is non-unique, but there are only finitely many possibilities, determined by the choice of the tile map. Let 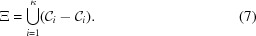
It is possible to consider a tile map 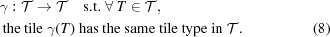
Then for any 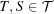, 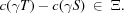
Let 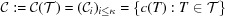
be a set of control points of the tiling 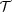 in 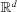. In what follows, if there is no confusion, we will use the same notation 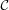 to mean 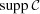.

For the main results of this paper, we need the property that 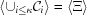 with 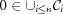. Under the assumption that ϕ is unimodular, this can be achieved by taking a proper control point set which comes from a certain tile map. We define the tile map as follows. It is known that there exists a finite patch 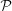 in a primitive substitution tiling which generates the whole tiling 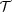 (Lagarias & Wang, 2003 ▸). Although it was defined with primitive substitution point sets by Lagarias & Wang (2003 ▸), it is easy to see that the same property holds for primitive substitution tilings. We call the finite patch 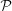 the *generating tile set*. When we apply the substitution infinitely many times to the generating tile set 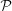, we obtain the whole substitution tiling. So there exists 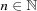 such that *n*th iteration of the substitution to the generating tile set covers the origin. We choose a tile *R* in a patch 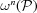 which contains the origin, where 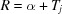 for some 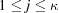. Then there exists a fixed tile 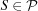 such that 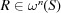. Replacing the substitution ω by 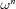, we can define a tile map γ so that 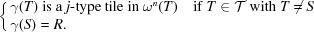
Then 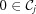 by the definition of the control point sets and so 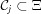. Notice that 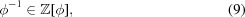
since ϕ is unimodular. From the construction of the tile map, we have 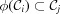 for any 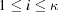. From (9), 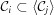 for any 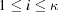. Hence 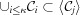. Thus 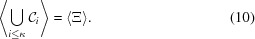

**Remark 2.4**

In the case of primitive unimodular irreducible one-dimensional Pisot substitution tilings, it is known that 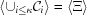 by choosing the left end points of the tiles as the control points (see Barge & Kwapisz, 2006 ▸; Sing, 2007 ▸).

### 2.4. Pure point spectrum and algebraic coincidence   

Let 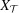 be the collection of tilings on 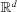 each of whose patches is a translate of a 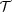-patch. In the case that 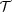 has FLC, we give a usual metric δ on the tilings in such a way that two tilings are close if there is a large agreement on a big region with small shift (see Schlottmann, 2000 ▸; Radin & Wolff, 1992 ▸; Lee *et al.*, 2003 ▸). Then 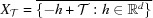
where the closure is taken in the topology induced by the metric δ. For non-FLC tilings, we can consider ‘local rubber topology’ on the collection of tilings (Müller & Richard, 2013 ▸; Lenz & Stollmann, 2003 ▸; Baake & Lenz, 2004 ▸; Lee & Solomyak, 2019 ▸) and obtain 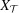 as the completion of the orbit closure of 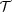 under this topology. For tilings with FLC, the two topologies coincide. In the case of either FLC or non-FLC tilings, we obtain a compact space 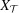. We have a natural action of 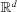 on the dynamical hull 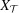 of 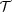 by translations and get a topological dynamical system 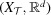. Let us assume that there is a unique ergodic measure μ in the dynamical system 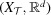 and consider the measure-preserving dynamical system 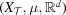. It is known that a dynamical system 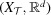 with a primitive substitution tiling 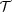 always has a unique ergodic measure (Solomyak, 1997 ▸; Lee *et al.*, 2003 ▸). We consider the associated group of unitary operators 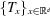 on 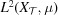: 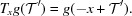
Every 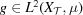 defines a function on 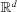 by 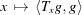. This function is positive definite on 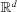, so its Fourier transform is a positive measure 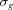 on 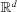 called the *spectral measure* corresponding to *g*. The dynamical system 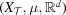 is said to have *pure discrete spectrum* if 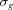 is pure point for every 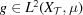. We also say that 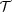 has pure discrete spectrum if the dynamical system 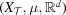 has pure discrete spectrum.

When we restrict discussion to primitive substitution tilings, we note that a tiling 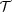 has pure discrete spectrum if and only if the control point set 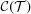 of the tiling 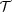 admits an algebraic coincidence (see Definition 2.5[Statement definition2.5]). So from now on when we assume pure discrete spectrum for 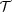, we can directly use the property of algebraic coincidence. We give the corresponding definition and theorem below.

**Definition 2.5**

Let 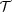 be a primitive substitution tiling on 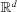 with an expansive map ϕ and 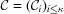 be a corresponding control point set. We say that 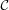 admits an *algebraic coincidence* if there exists 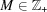 and 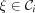 for some 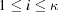 such that 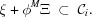
Here recall from (7) that 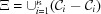.

Note that, if the algebraic coincidence is assumed, then for some 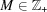


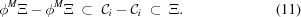

**Theorem 2.6**

[Theorem 3.13 (Lee, 2007 ▸)] Let 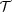 be a primitive substitution tiling on 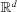 with an expansive map ϕ and 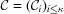 be a corresponding control point set. Suppose that all the eigenvalues of ϕ are algebraic integers. Then 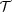 has pure discrete spectrum if and only if 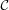 admits an algebraic coincidence.

The above theorem is stated with FLC by Lee (2007 ▸). But from Lemma 4.1 and Proposition 4.2, pure discrete dynamical spectrum of 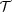 implies the Meyer property of the control point set 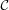. All Meyer sets have FLC. So it is a consequence of pure discrete dynamical spectrum. On the other hand, the algebraic coincidence implies that 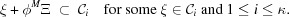
This means that 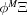 is uniformly discrete and thus Ξ is uniformly discrete. From Moody (1997 ▸), we obtain that 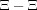 is uniformly discrete. For any 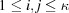, 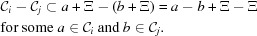
Hence 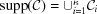 is a Meyer set (Moody, 1997 ▸). Thus it is not necessary to assume FLC here. There is a computable algorithm to check the algebraic coincidence in a primitive substitution tiling (Akiyama & Lee, 2011 ▸).

### 2.5. Cut-and-project scheme   

We give definitions for a CPS and model sets constructed with 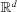 and a locally compact Abelian group.

**Definition 2.7**

A *cut-and-project scheme* (CPS) consists of a collection of spaces and mappings as follows: 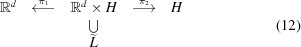
where 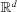 is a real Euclidean space, *H* is a locally compact Abelian group, 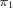 and 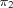 are the canonical projections, 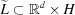 is a lattice, *i.e.* a discrete subgroup for which the quotient group 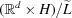 is compact, 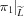 is injective and 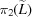 is dense in *H*. For a subset 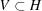, we denote 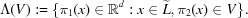 A *model set* in 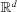 is a subset 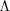 of 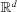 of the form 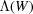, where 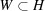 has non-empty interior and compact closure. The model set 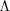 is *regular* if the boundary of *W*

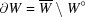
is of (Haar) measure 0. We say that 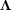 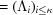 is a *model* κ-*set* (respectively, *regular model* κ-*set*) if each 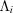 is a model set (respectively, regular model set) with respect to the same CPS. Especially when *H* is a Euclidean space, we call the model set Λ a *Euclidean model set* (see Baake & Grimm, 2013 ▸).

## 3. Cut-and-project scheme on substitution tilings   

Throughout the rest of the paper, we assume that ϕ is diagonalizable, the eigenvalues of ϕ are algebraically conjugate with the same multiplicity, since the structure of a module generated by the control points is known only under this assumption (Lee & Solomyak, 2012 ▸).

Let 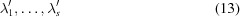
be the distinct real eigenvalues of ϕ and 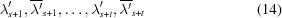
be the distinct complex eigenvalues of ϕ. By the above assumption, all these eigenvalues appear with the same multiplicity, which we will denote by *J*. Recall that ϕ is assumed to be diagonalizable over 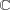. For a complex eigenvalue λ of ϕ, the 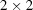 diagonal block
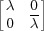
is similar to a real 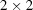 matrix 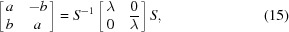
where 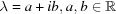, and
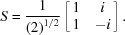
Since ϕ is diagonalizable, by eventually changing the basis in 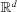, we can assume without loss of generality that 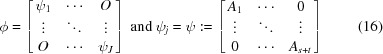
where 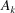 is a real 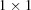 matrix for 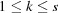, a real 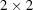 matrix of the form
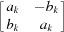
for 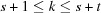, and 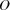 is the 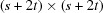 zero matrix, and 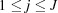.

Let 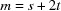. Note that *m* is the degree of the minimal polynomial of ϕ over 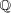. For each 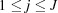, let 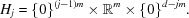
Further, for each 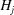 we have the direct sum decomposition
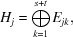
such that each 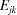 is 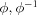-invariant and 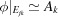, identifying 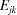 with 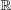 or 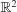.

Let 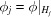.

Let 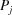 be the canonical projection of 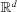 onto 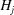 such that 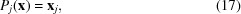
where 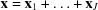 and 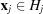 with 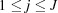.

We define 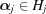 such that for each 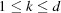, 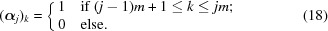
We recall the following theorem for the module structure of the control point sets. From Lemma 6.1 (Lee & Solomyak, 2012 ▸), we can readily obtain the property:^1^

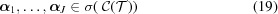
which is used in the proof of Lemma 5.2. So we state Theorem 4.1 (Lee & Solomyak, 2012 ▸) in the following form. Let
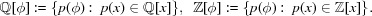

**Theorem 3.1**

[Theorem. 4.1 (Lee & Solomyak, 2012 ▸)] Let 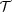 be a repetitive primitive substitution tiling on 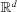 with an expansion map ϕ. Assume that 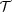 has FLC, ϕ is diagonalizable, and all the eigenvalues of ϕ are algebraically conjugate with the same multiplicity *J*. Then there exists an isomorphism 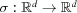 such that 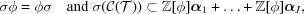
where 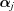, 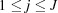, are given in (18), and 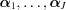 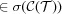.

Since ϕ is a block diagonal matrix as shown in (16), we can note that 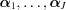 are linearly independent over 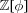.

A tiling 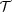 is said to be *rigid* if 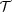 satisfies the result of Theorem 3.1[Statement theorem3.1]; that is to say, there exists a linear isomorphism 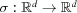 such that 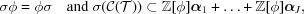
where 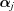, 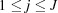, are given in (18). One can find an example of a non-FLC tiling that the rigidity property fails in (Frank & Robinson, 2008 ▸; Lee & Solomyak, 2019 ▸).

### 3.1. Construction of a cut-and-project scheme   

Consider that ϕ is unimodular and diagonalizable, all the eigenvalues of ϕ are algebraic integers and algebraically conjugate with the same multiplicity *J*, and 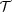 is rigid. Since ϕ is an expansion map and unimodular, there exists at least one other algebraic conjugate other than eigenvalues of ϕ. Under this condition, we construct a CPS with a Euclidean internal space. In the case of multiplicity 1, the CPS was first introduced in Lee *et al.* (2018 ▸). For earlier development, see Siegel & Thuswaldner (2009 ▸).

It is known that if ϕ is a diagonalizable expansion map of a primitive substitution tiling with FLC, every eigenvalue of ϕ is an algebraic integer (Kenyon & Solomyak, 2010 ▸). So it is natural to assume that all the eigenvalues of ϕ are algebraic integers in the assumption. In (16), suppose that the minimal polynomial of ψ over 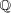 has *e* number of real roots and *f* number of pairs of complex conjugate roots. Recall that 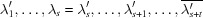
are distinct eigenvalues of ϕ from (13) and (21). Let us consider the roots in the following order: 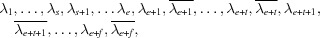
for which 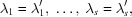


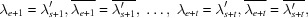
where 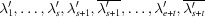 are the same as in (13) and (14).

Let 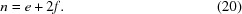
We consider a space where the rest of the roots of the minimal polynomial of ψ other than the eigenvalues of ψ lie. Using similar matrices as in (15) we can consider the space as a Euclidean space. Let 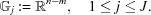
For 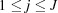, define a 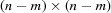 matrix 
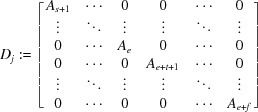
where 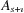 is a real 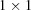 matrix with the value 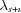 for 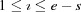, and 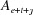 is a real 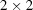 matrix of the form
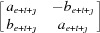
for 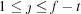. Notice that ϕ and ψ have the same minimal polynomial over 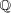, since ϕ is the diagonal matrix containing *J* copies of ψ. Let us consider now the following algebraic embeddings: 
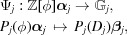
where 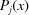 is a polynomial over 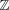 and 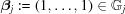. Note that 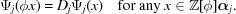
Now we can define a map 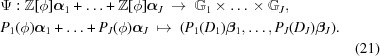
Since 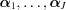 are linearly independent over 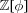, the map Ψ is well defined. Thus 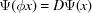 for 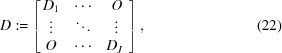
where 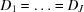. Let 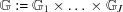.

Let us construct a CPS: 
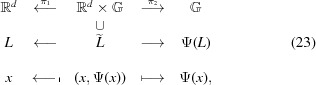
where 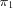 and 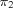 are canonical projections, 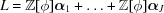
and 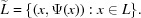
It is easy to see that 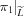 is injective. We shall show that 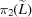 is dense in 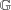 and 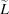 is a lattice in 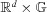. We note that 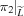 is injective, since Ψ is injective. Since ϕ commutes with the isomorphism σ in Theorem 3.1[Statement theorem3.1], we may identify 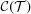 and its isomorphic image. Thus, from Theorem 3.1[Statement theorem3.1], 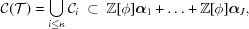
where 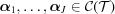. Note that for any 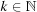 and 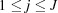, 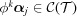. So we can note that 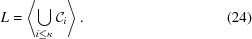

**Lemma 3.2**

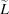
 is a lattice in 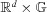.

**Proof**

By the Cayley–Hamilton theorem, there exists a monic polynomial 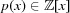 of degree *n* such that 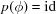. Thus every element of 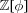 is expressed as a polynomial of ϕ of degree 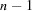 with integer coefficients where the constant term is identified with a constant multiple of the identity matrix. Therefore *L* is a free 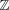-module of rank *nJ*. Notice that *L* and 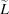 are isomorphic 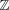-modules so that 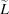 is also a free 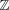-module of rank *nJ* on 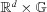. Let us define 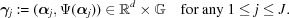
Then, in fact, for any 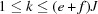, 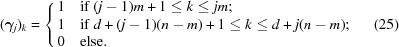
Define also 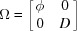
which is a linear map on 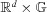. Note that 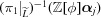 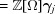 and 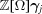 is isomorphic to the image of 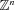 by multiplication of the 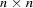 matrix 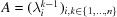. Since *A* is non-degenerate by the Vandermonde determinant, 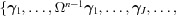 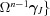 forms a basis of 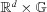 over 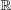. Thus 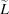 is a lattice in 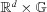.

□

**Lemma 3.3**

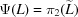
 and 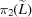 is dense in 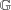.

**Proof**

For simplicity, we prove the totally real case, *i.e.* 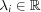 for all *i*. Since the diagonal blocks of ϕ are all the same, it is enough to show that 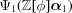 is dense in 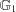. By Theorem 24 (Siegel, 1989 ▸), 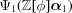 is dense in 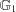 if
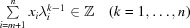
implies 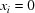 for 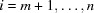. The condition is equivalent to 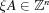 with 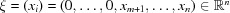 in the terminology of Lemma 3.2[Statement lemma3.2]. Multiplying by the inverse of *A*, we see that the entries of ξ must be Galois conjugates. As ξ has at least one zero entry, we obtain 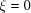 which shows 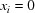 for 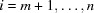. In fact, this discussion is using the Pontryagin duality that the 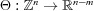 has a dense image if and only if its dual map 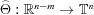 is injective [see also Meyer (1972 ▸, ch. II, Section 1), Iizuka *et al.* (2009 ▸), Akiyama (1999 ▸)]. The case with complex conjugates is similar.

□

Now that we have constructed the CPS (23), we would like to introduce a special projected set 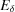 which will appear in the proofs of the main results in Section 5. For 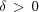, we define 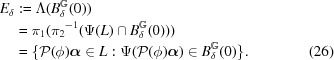
In the following lemma, we find an adequate window for a set 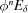 and note that 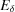 is a Meyer set.

**Lemma 3.4**

For any 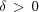 and 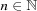, if 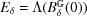, then 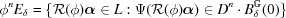
and 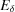 forms a Meyer set.

**Proof**

Note that 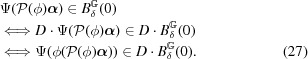
Notice that if ϕ is unimodular, then 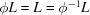 and 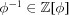. Thus 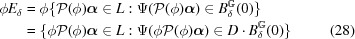


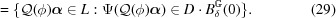
It is easy to see that the set in (28) is contained in the set in (29). The inclusion for the other direction is due to the fact that 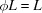 and 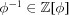. Hence for any 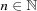, 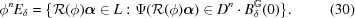
Since (23) is a CPS and 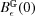 is bounded, 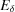 forms a Meyer set for each 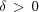 (see Moody, 1997 ▸).

□

<!?tpb=-12pt>

## 4. Pure discrete spectrum, Meyer set and Pisot family  

**Lemma 4.1**

[Lemma 4.10 (Lee & Solomyak, 2008 ▸)] Let 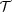 be a tiling on 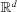. Suppose that 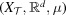 has pure discrete dynamical spectrum. Then the eigenvalues for the dynamical system 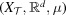 span 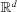.

**Proposition 4.2**

[Proposition 6.6 (Lee & Solomyak, 2019 ▸)] Let 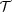 be a primitive substitution tiling on 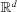 with an expansion map ϕ. Suppose that all the eigenvalues of ϕ are algebraic integers. Assume that the set of eigenvalues of 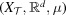 is relatively dense. Then 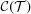 is a Meyer set.

We note that ‘repetitivity’ is not necessary for Proposition 4.2[Statement proposition4.2]. Under the assumption that 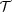 is a primitive substitution tiling on 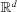, the following implication holds: 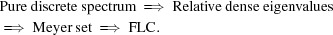

**Definition 4.3**

A set of algebraic integers 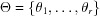 is a *Pisot family* if for any 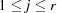, every Galois conjugate γ of 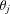, with 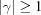, is contained in Θ. For 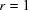, with 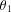 real and 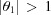, this reduces to 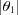 being a real Pisot number, and for 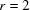, with 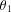 non-real and 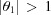, to 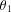 being a complex Pisot number.

Under the assumption of rigidity of 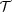, we can derive the following proposition from Lemma 5.1 (Lee & Solomyak, 2012 ▸) without additionally assuming repetitivity and FLC.

**Proposition 4.4**

[Lemma 5.1 (Lee & Solomyak, 2012 ▸)] Let 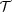 be a primitive substitution tiling on 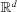 with a diagonalizable expansion map ϕ. Suppose that all the eigenvalues of ϕ are algebraic conjugates with the same multiplicity and 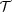 is rigid. Then if the set of eigenvalues of 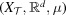 is relatively dense, then the set of eigenvalues of ϕ forms a Pisot family.

## 5. Main result   

We consider a primitive substitution tiling on 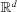 with a diagonalizable expansion map ϕ. Suppose that all the eigenvalues of ϕ are algebraically conjugate with the same multiplicity *J* and 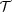 is rigid. Additionally we assume that there exists at least one algebraic conjugate λ of eigenvalues of ϕ for which 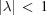. Recall that 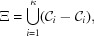
where 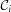 is the set of control points of tiles of type *i* and 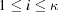. By the choice of the control point set in (10), we note that 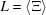.

**Lemma 5.1**

Assume that the set of eigenvalues of ϕ is a Pisot family. Then 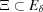 for some 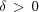, where 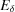 is given in (26).

**Proof**

Since we are interested in Ξ which is a collection of translation vectors, the choice of control point set 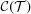 does not really matter. So we use the tile map (8) which sends a tile to the same type of tiles in 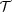. From Lemma 4.5 (Lee & Solomyak, 2008 ▸), for any 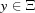, 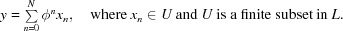
Since ϕ is an expansive map and satisfies the Pisot family condition, the maps 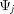 and Ψ are defined with all the algebraic conjugates of eigenvalues of ϕ whose absolute values are less than 1. Thus 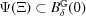 for some 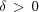. From the definition of 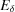 in (26), 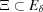.

□

**Lemma 5.2**

Assume that 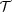 has pure discrete spectrum. Then for any 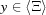, there exists 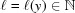 such that 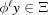.

**Proof**

Note from (24) that for any 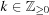 and 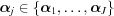, 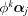 is contained in Ξ. Recall that 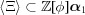 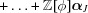, where 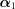, 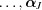 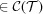. So any element 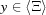 is a linear combination of 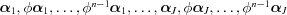 over 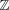. Applying (11) many times if necessary, we get that for any 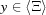, 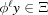 for some 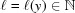.

□

**Proposition 5.3**

Let 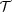 be a primitive substitution tiling on 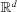 with an expansion map ϕ. Under the assumption of the existence of CPS (23), if 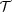 has pure discrete spectrum, then there exists 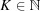 such that 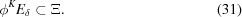

**Proof**

We first prove that there exists a finite set *F* such that for all 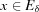, 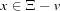 for some 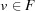. This can be obtained directly from Lemma 5.5.1 (Strungaru, 2017 ▸; Baake & Grimm, 2017 ▸), but for the reader’s convenience we give the proof here. Note that 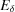 is a Meyer set and 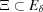 for some 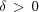. Since Ξ is relatively dense, for any 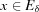, there exists 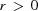 such that 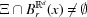. From the Meyer property of 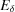, the point set configurations 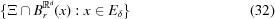
are finite up to translation elements of 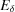. We should note that if 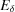 has FLC but not the Meyer property, the property (32) may not hold. Let
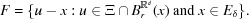
Then 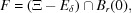


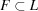
, and *F* is a finite set. Thus for any 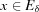, 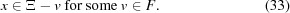
From Lemma 5.2[Statement lemma5.2] and 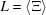, for any 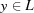, there exists 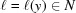 such that 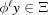. By the pure discrete spectrum of 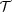 and (11), there exists 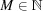 such that 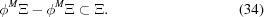
Applying the containment (34) finitely many times, we obtain that there exists 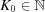 such that 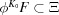. Hence together with (33), there exists 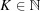 such that 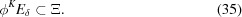

□

<!?tpb=-12pt>

In order to discuss model sets and compute the boundary measures of their windows for substitution tilings, we need to introduce 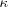-set substitutions for substitution Delone sets which represent the substitution tilings.

**Definition 5.4**

For a substitution Delone κ-set 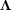 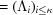 satisfying (2), define a matrix 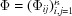 whose entries are finite (possibly empty) families of linear affine transformations on 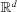 given by 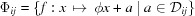 . We define 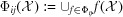 for 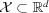. For a κ-set 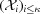 let 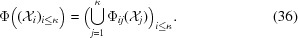
Thus 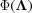 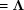 by definition. We say that Φ is a κ-*set substitution*. Let 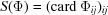
be a *substitution matrix* corresponding to Φ. This is analogous to the substitution matrix for a tile-substitution.

Recall that there exists a finite generating set 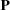 such that 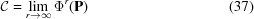
from Lagarias & Wang (2003 ▸), Lee *et al.* (2003 ▸). If the finite generating set 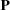 consists of a single element, we say that 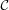 is *generated from one point*. Since 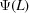 is dense in 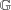 by Lemma 3.3[Statement lemma3.3], we have a unique extension of Φ to a κ-set substitution on 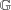 in the obvious way; if 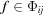 for which 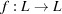, 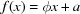, we define 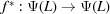, 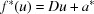, *D* is given in (22), and 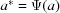. Since 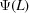 is dense in 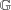, we can extend the mapping 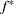 to 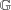. If there is no confusion, we will use the same notation 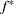 for the extended map.

Note that, by the Pisot family condition on ϕ, there exists some 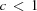 such that 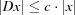 for any 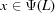. This formula defines a mapping on 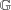 and 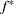 is a contraction on 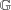. Thus a κ-set substitution Φ determines a multi-component iterated function system 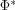 on 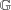. Let 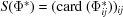 be a *substitution matrix* corresponding to 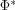. Defining the compact subsets 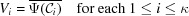
and using (36) and the continuity of the mappings, we have 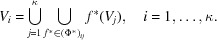 This shows that 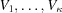 are the unique attractor of 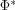.

**Remark 5.5**

From Proposition 4.4 (Lee, 2007 ▸), if 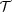 has pure discrete spectrum, then there exists 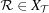 such that the control point set 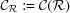 of the tiling 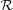 satisfies 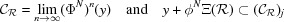
for some 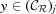, 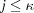 and 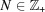. Note that 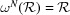. Let 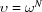. We can consider a *r*th-level supertiling 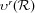 of 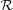. Note that there exists an *r*th-level supertile 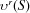 in 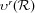 containing the origin in the support which contains the tile 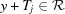. Redefining the tile map for the control points of this supertiling so that the control point of the *r*th-level supertile 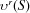 is at the origin, we can build a substitution tiling 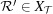 for which algebraic coincidence occurs at the origin. So rewriting the substitution if necessary, we can assume that 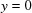. With this assumption, we get the following proposition.

**Proposition 5.6**

Let 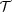 be a primitive substitution tiling on 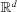 with a diagonalizable expansion map ϕ which is uni­modular. Suppose that all the eigenvalues of ϕ are algebraic conjugates with the same multiplicity and 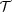 is rigid. Suppose that 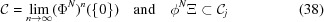
for some 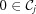, 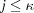 and 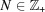. Assume that CPS (23) exists. Then each point set 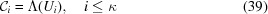
is a Euclidean model set in CPS (23) with a window 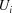 in 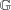 which is open and pre-compact.

**Proof**

For each 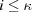 and 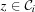, there exists 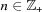 such that 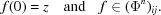
From 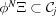, 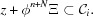
By Theorem 2.6[Statement theorem2.6] and Proposition 5.3[Statement proposition5.3], there exists 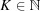 such that 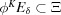. Thus 
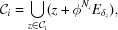
where 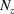 depends on *z*. From the equality of (30), we let 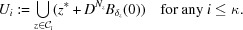
Then 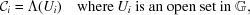
for any 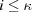.

From Lemma 5.1[Statement lemma5.1], 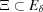 for some 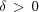. Thus 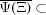 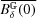. Since 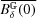 is compact, 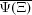 is compact. Thus 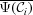 is compact.

□

We can assume that the open window 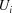 in (39) is the maximal element satisfying (39) for the purpose of proving the following proposition. In this proposition, we show that the control point set 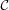 is a regular model set using Keesling’s argument (Keesling, 1999 ▸).

**Proposition 5.7**

Let 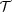 be a repetitive primitive substitution tiling on 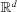 with a diagonalizable expansion map ϕ which is unimodular. Suppose that all the eigenvalues of ϕ are algebraic conjugates with the same multiplicity and 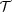 is rigid. Under the assumption of the existence of CPS (23), if 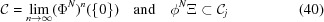
where 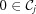, 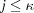 and 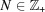, then each Euclidean model set 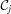, 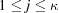 has a window with boundary measure zero in the Euclidean internal space 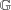 of CPS (23).

**Proof**

Let us define 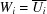, where 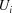 is the maximal open set in 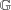 satisfying (39). From the assumption of (40), we first note that ϕ fulfils the Pisot family condition from Theorem 2.6[Statement theorem2.6] and Proposition 4.4[Statement proposition4.4]. For every measurable set 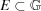 and for any 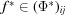 with 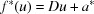, 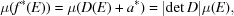
where μ is a Haar measure in 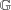 and *D* is the contraction as given in (22). Note that 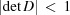. In particular, 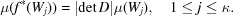
We have attractors 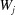
’s satisfying 
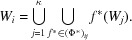
Let us denote 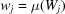 for 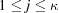 and 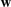 = 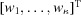. Then for any 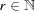, 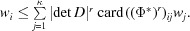
Note here that for any 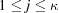, 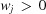 follows from the fact that 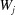 has a non-empty interior. Thus 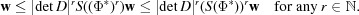
Note from Lagarias & Wang (2003 ▸) that the Perron eigenvalue of 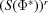 is 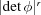. From the unimodular condition of ϕ, 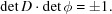
Since 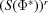 is primitive, from Lemma 1 (Lee & Moody, 2001 ▸) 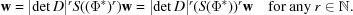
By the positivity of 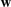 and 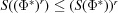, 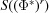 = 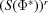.

Recall that for any 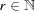, 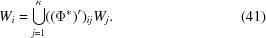
From (3), for any 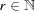, 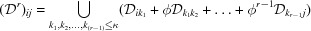
and 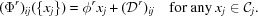
Note that 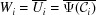 and 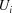 is a non-empty open set. As 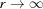, 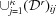 is dense in 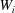. Since 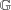 is a Euclidean space, we can find a non-empty open set 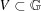 such that 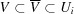. So there exist 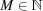 and 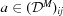 such that 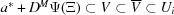. Since 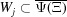, 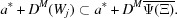
Thus there exists 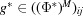 such that 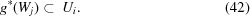
Hence 
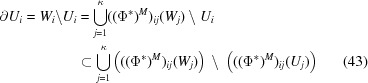


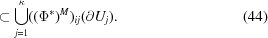
The inclusion (43) is followed by the maximal choice of an open set 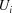. Let
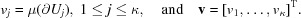
Then 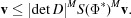
From (42), we observe that not all functions in 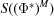 are used for the inclusion (44). Thus there exists a matrix 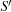 for which 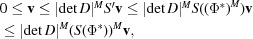
where 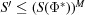 and 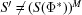. If 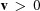, again from Lemma 1 (Lee & Moody, 2001 ▸), 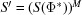. This is a contradiction to (42). Therefore 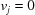 for any 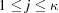.

□

The regularity property of model sets can be shared for all the elements in 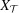. One can find the earliest result of this property in the work of Schlottmann (2000 ▸) and the further development in the work of Baake *et al.* (2007 ▸), Keller & Richard (2019 ▸) and Lee & Moody (2006 ▸). We state the property [Proposition 4.4 (Lee & Moody, 2006 ▸)] here.

**Proposition 5.8**

(Schlottmann, 2000 ▸; Baake *et al.*, 2007 ▸; Keller & Richard, 2019 ▸; Lee & Moody, 2006 ▸) Let 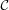 be a Delone κ-set in 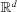 for which 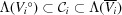 where 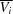 is compact and 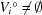 for 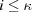 with respect to some CPS. Then for any 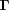 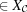, there exists 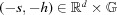 so that 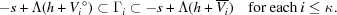

From the assumption of pure discrete spectrum and Remark 5.5[Statement enun5.5], we can observe that the condition (40) is fulfilled in the following theorem.

**Theorem 5.9**

Let 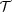 be a repetitive primitive substitution tiling on 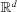 with a diagonalizable expansion map ϕ which is unimodular. Suppose that all the eigenvalues of ϕ are algebraically conjugate with the same multiplicity. If 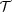 has pure discrete spectrum, then each control point set 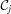, 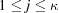, is a regular Euclidean model set in CPS (23).

**Proof**

Under the assumption of pure discrete spectrum, we know that 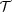 has FLC from the work of Lee & Solomyak (2019 ▸) and ϕ fulfils the Pisot family condition (Lee & Solomyak, 2012 ▸). From Theorem 3.1[Statement theorem3.1], we know that 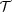 is rigid. Since ϕ is unimodular, there exists at least one algebraic conjugate λ of eigenvalues of ϕ for which 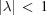. Thus we can construct the CPS (23) with a Euclidean internal space. Since 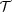 has pure discrete spectrum and is repetitive, we can find a substitution tiling 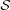 in 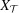 such that 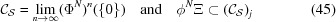
where 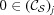, 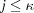 and 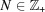. The claim follows from Propositions 5.3[Statement proposition5.3], 5.7[Statement proposition5.7] and 5.8[Statement proposition5.8].

□

**Corollary 5.10**

Let 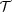 be a repetitive primitive substitution tiling on 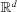 with a diagonalizable expansion map ϕ which is unimodular. Suppose that all the eigenvalues of ϕ are algebraically conjugate with the same multiplicity. Then 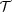 has pure discrete spectrum if and only if each control point set 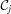, 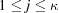, is a regular Euclidean model set in CPS (23).

**Proof**

It is known that any regular model sets have pure discrete spectrum in quite a general setting (Schlottmann, 2000 ▸). Together with Theorem 5.9[Statement theorem5.9], we obtain the equivalence between pure discrete spectrum and regular model set in substitution tilings.

□

The next example shows that the unimodularity of ϕ is necessary.

**Example 5.11**

Let us consider an example of non-unimodular substitution tiling which is studied by Baake *et al.* (1998 ▸). This example is proven to be a regular model set in the setting of a CPS constructed by Baake *et al.* (1998 ▸) with the help of 2-adic embedding. In our setting of CPS (23), we show that this example cannot provide a model set, since we are only interested in the Euclidean window in this paper.

The substitution matrix of the primitive two-letter substitution 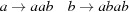
has the Perron–Frobenius eigenvalue 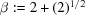 which is a Pisot number but non-unimodular. We can extend the letter *a* to the right-hand side by the substitution and the letter *b* to the left-hand side. So we can get a bi-infinite sequence fixed under the substitution. A geometric substitution tiling arising from this substitution can be obtained by replacing symbols *a* and *b* in this sequence by the intervals of length 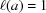 and 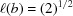. Then we have the following tile-substitution ω,
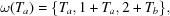


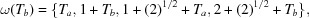
where 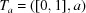 and 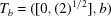. Considering return words 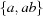 for *a*, and 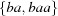 for *b*, we can check 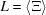. We choose left end points 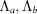 of corresponding intervals as the set of control points. Then they satisfy
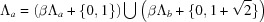


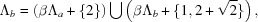
by Lagarias–Wang duality (Lagarias & Wang, 2003 ▸). Applying the Galois conjugate κ which sends 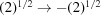, we obtain a generalized iterated function system
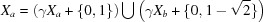


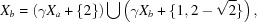
with 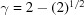, 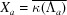 and 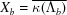. We can easily confirm that
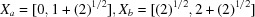
are the unique attractors of this iterated function system. Since 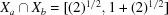 contains an inner point, it is unable to distinguish them by any window in this setting.

## 6. Further study   

We have mainly considered unimodular substitution tilings in this paper. Example 5.11[Statement example5.11] shows a case of non-unimodular substitution tiling which is studied by Baake *et al.* (1998 ▸). It cannot be a Euclidean model set in the cut-and-project scheme (23) that we present in this paper, but it is proven to be a regular model set in the setting of a cut-and-project scheme constructed in the work of Baake *et al.* (1998 ▸), which suggests non-unimodular tilings require non-Archimedean embeddings to construct internal spaces. It is an intriguing open question to construct a concrete cut-and-project scheme in this case.

## Footnotes

1 This fact is stated in a slightly different way in the work of Lee & Solomyak (2012 ▸).
